# Up-regulation of 5-lipoxygenase by inhibition of cathepsin G enhances TRAIL-induced apoptosis through down-regulation of survivin

**DOI:** 10.18632/oncotarget.22508

**Published:** 2017-11-20

**Authors:** Seon Min Woo, Kyoung-Jin Min, Seung Un Seo, Shin Kim, Jong-Wook Park, Dae Kyu Song, Hyun-Shik Lee, Sang Hyun Kim, Taeg Kyu Kwon

**Affiliations:** ^1^ Department of Immunology, School of Medicine, Keimyung University, Dalseo-Gu, Daegu 704-701, South Korea; ^2^ Department of Physiology, School of Medicine, Keimyung University, Dalseo-Gu, Daegu 704-701, South Korea; ^3^ KNU-Center for Nonlinear Dynamics, School of Life Sciences, BK21 Plus KNU Creative BioResearch Group, College of Natural Sciences, Kyungpook National University, Daegu 41566, South Korea; ^4^ Department of Pharmacology, School of Medicine, Kyungpook National University, Daegu, South Korea

**Keywords:** cathepsin G, TRAIL, survivin, ROS, 5-LOX

## Abstract

Cathepsin G is a serine protease secreted from activated neutrophils, it has important roles in inflammation and immune response. Moreover, cathepsin G promotes tumor cell-cell adhesion and migration in cancer cells. In this study, we investigated whether inhibition of cathepsin G could sensitize TRAIL-mediated apoptosis in cancer cells. An inhibitor of cathepsin G [Cathepsin G inhibitor I (Cat GI); CAS 429676-93-7] markedly induced TRAIL-mediated apoptosis in human renal carcinoma (Caki, ACHN, and A498), lung cancer (A549) and cervical cancer (Hela) cells. In contrast, combined treatment with Cat GI and TRAIL had no effect on apoptosis in normal cells [mesangial cell (MC) and human skin fibroblast (HSF)]. Cat GI induced down-regulation of survivin expression at the post-translational level, and overexpression of survivin markedly blocked apoptosis induced by combined treatment with Cat GI plus TRAIL. Interestingly, Cat GI induced down-regulation of survivin via 5-lipoxygenase (5-LOX)-mediated reactive oxygen species (ROS) production. Inhibition of 5-LOX by gene silencing (siRNA) or a pharmacological inhibitor of 5-LOX (zileuton) markedly attenuated combined treatment-induced apoptosis. Taken together, our results indicate that inhibition of cathepsin G sensitizes TRAIL-induced apoptosis through 5-LOX-mediated down-regulation of survivin expression.

## INTRODUCTION

Cathepsin G, a serine protease, is mainly expressed in antigen presenting cells (APC) such as primary human B cells, myeloid dendritic cells and murine microglia [1–4], and secreted by activated neutrophils and human monocyte [5]. Inhibition of cathepsin G reduces antigen processing and modulates T cell response in primary dendritic cells [6]. In addition, cathepsin G modulates cell death in multiple cells. For examples, cathepsin G promotes protease-activated receptor-independent apoptosis and protein-tyrosine phosphatase SHP2-mediated anoikis in caridiomyocyte [7, 8], and inhibition of cathepsin G by mycobacterium tuberculosis prevents caspase-1-dependent pyroptosis in macrophages [9]. Recently, roles for cathepsin G in cancer cells have been reported [10–13]. Treatment with cathepsin G induces cell migration, insulin-like growth factor-1 signaling-dependent multicellular aggregation and E-cadherin-mediated cell-cell adhesion in human breast cancer MCF-7 cells [10–13]. Furthermore, cathepsin G-induced transforming growth factor (TGF)-β signaling promotes angiogenesis through up-regulation of VEGF and MCP-1 expression at the tumor-bone interface [14]. However, the role of cathepsin G in cancer progression and cancer cell death is not fully understood.

Tumor necrosis factor (TNF)-related apoptosis-inducing ligand (TRAIL) selectively triggers apoptosis in various cancer cells but not in normal cells [15]. TRAIL binds death receptor (DR) and then forms death-inducing signal complex (DISC) with FAS-associated protein death domain (FADD) and caspase-8. The process of TRAIL-induced apoptosis categorizes two signaling pathways. In extrinsic pathway, activation of caspase-8 by DISC directly induces caspase-3-dependent apoptosis. On the other hand, in intrinsic pathway, truncation of BH3-interacting domain death agonist (BID) by caspase-8 is translocated to the mitochondria, and consequently increases caspase-9-dependent apoptosis. However, many cancer cells have resistance to TRAIL [16, 17]. Cancer cells overexpress anti-apoptotic proteins [anti-apoptotic Bcl-2 family and inhibitor of apoptosis proteins (IAPs)] and down-regulate DR expression [18–21]. Therefore, identification of effective sensitizer is one approach to overcome TRAIL resistance in cancer cells.

Reactive oxygen species (ROS) are endogenously generated by a variety of cellular oxidative processes, including the mitochondrial respiratory chain, NADPH oxidases (NOX), xanthine oxidases (XO), lipoxygenases (LOX) and cyclooxygenases (COX). Production of intracellular ROS plays major roles a various signaling pathways that induce apoptosis, senescence and differentiation in tumor cells [22, 23]. 5-LOX, the enzyme of arachidonic acid (AA) metabolism, is highly expressed in various cancer cells such as prostate [24], colon [25], renal [26], and pancreas [27] carcinoma cells. 5-LOX-mediated ROS generation is involved in inflammation, proliferation and angiogenesis [28, 29]. In addition, docosahexaenoyl ethanolamide (DHEA) and N-arachidonoyl-L-alanine (NALA) inhibits proliferation through 5-LOX-mediated ROS generation in head and neck squamous cell carcinoma [30].

In this study, we investigated whether inhibition of cathepsin G sensitizes TRAIL-mediated apoptosis. We found that Cat GI induces 5-LOX mediated-ROS that enhances TRAIL-induced apoptosis through down-regulation of survivin expression. These results suggest that inhibition of cathepsin G could be a novel sensitizer against TRAIL.

## RESULTS

### Cathepsin G inhibitor I sensitizes TRAIL-mediated apoptosis in human renal carcinoma Caki cells

**Figure 1:**
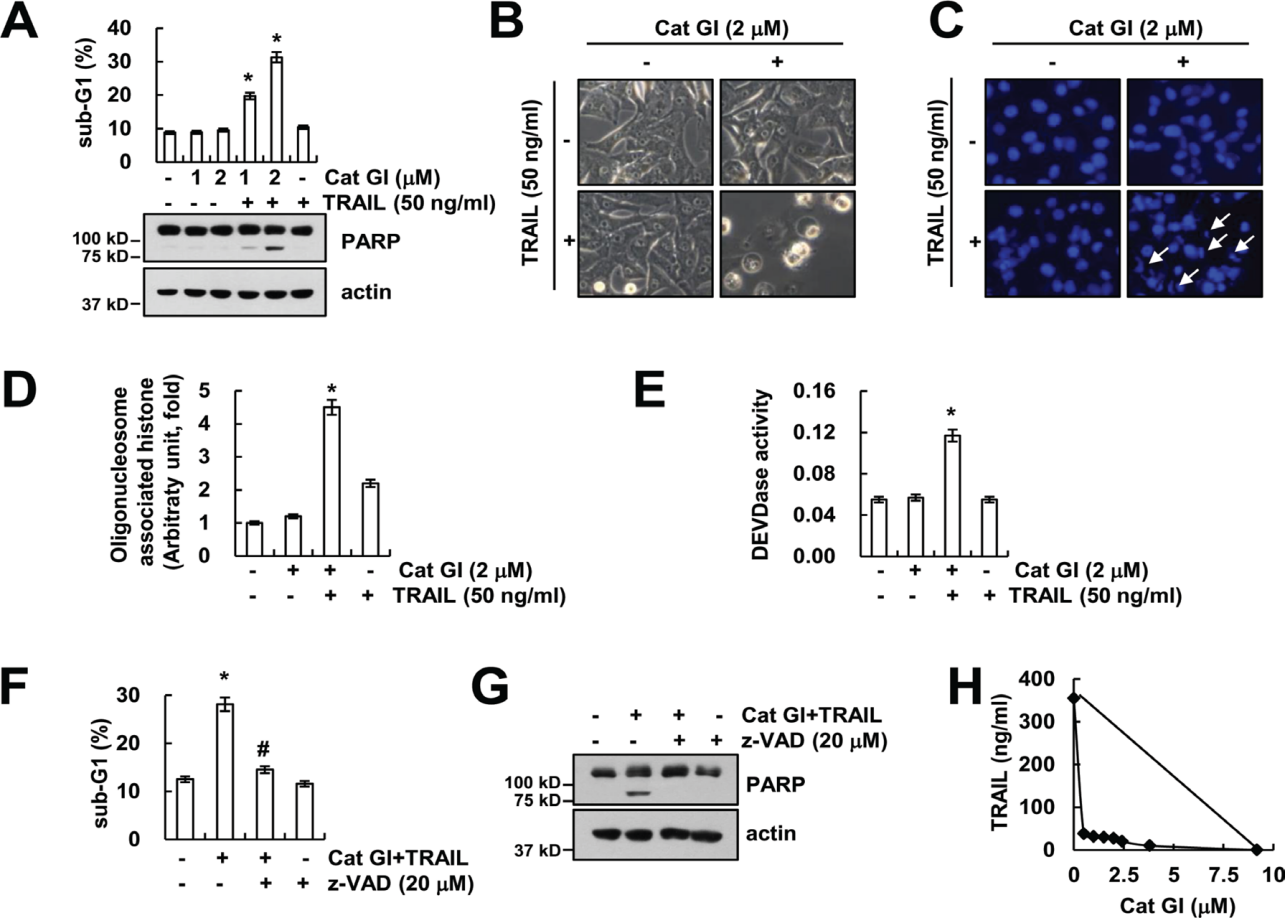
Cathepsin G inhibitor (Cat GI) sensitizes Caki cells to TRAIL-induced apoptosis (**A**) Caki cells were treated with 50 ng/ml TRAIL in the presence or absence of the indicated concentrations of Cat GI for 24 h. The level of apoptosis was analyzed by the sub-G1 population using flow cytometry. The protein expression levels of PARP and actin were determined by western blotting. The level of actin was used as a loading control. (**B**–**E**) Caki cells were treated with 50 ng/ml TRAIL in the presence or absence of 2 µM Cat GI for 24 h. The cell morphology was examined using interference light microscopy (B). The condensation and fragmentation of the nuclei were detected by 4′,6′-diamidino-2-phenylindole staining (C). The cytoplasmic histone-associated DNA fragments were determined by a DNA fragmentation detection kit (D). Caspase activities were determined with colorimetric assays using caspase-3 (DEVDase) assay kits (E). (**F**–**G**) Caki cells were treated with 2 µM Cat GI plus 50 ng/ml TRAIL for 24 h in the presence or absence of 20 µM z-VAD-fmk (z-VAD). The level of apoptosis was analyzed by the sub-G1 population using flow cytometry (F). The protein expression levels of PARP and actin were determined by western blotting. The level of actin was used as a loading control (G). (**H**) Isoboles were obtained by plotting the combined concentrations of each drug required to produce 50% cell death. The straight line connecting the IC_50_ values obtained for the two agents when applied alone corresponded to the addition of their independent effects. Values below this line indicate synergy, whereas values above this line indicate antagonism. The values in A, D, E and F represent the mean ± SD from three independent samples; ^*^p < 0.01 compared to the control. ^#^*p* < 0.01 compared to the combined treatment with Cat GI and TRAIL.

Cathepsin G has important role in tumor development and metastasis [10–14]. Therefore, we investigated whether inhibition of cathepsin G could have anti-cancer effects in human renal carcinoma Caki cells. First, we examined the effect of cathepsin G inhibitor I (Cat GI) on TRAIL (sub-toxic dosage)-induced apoptosis. Treatment with Cat GI alone and TRAIL alone did not induce apoptosis, but combined treatment with Cat GI and TRAIL markedly increased apoptosis and cleavage of PARP (Figure 1A). We showed typical apoptotic morphologies and chromatin condensation in Cat GI plus TRAIL-treated cells (Figure 1B and 1C). Moreover, combined treatment with Cat GI and TRAIL induced DNA fragmentation (Figure 1D). Next, we examined whether activation of caspase is involved in Cat GI plus TRAIL-induced apoptosis. As shown in Figure 1E, treatment with Cat GI alone or TRAIL alone did not increase caspase-3 activity, but combined treatment with Cat GI and TRAIL induced caspase-3 activation. Furthermore, pretreatment with z-VAD-fmk, the pan-caspase inhibitor, completely inhibited Cat GI plus TRAIL-induced apoptosis and PARP cleavage (Figure 1F and 1G). We investigated whether co-treatment with Cat GI and TRAIL has synergistic effects using isobologram analysis. Combined treatment with various concentrations of Cat GI and TRAIL indicated synergistic effects (Figure 1H). Therefore, these results indicate that inhibition of cathepsin G sensitizes TRAIL-induced caspase-dependent apoptosis in human renal carcinoma cells.

### Effect of combined treatment with Cat GI plus TRAIL on apoptosis in other cancer cells and normal cells

**Figure 2:**
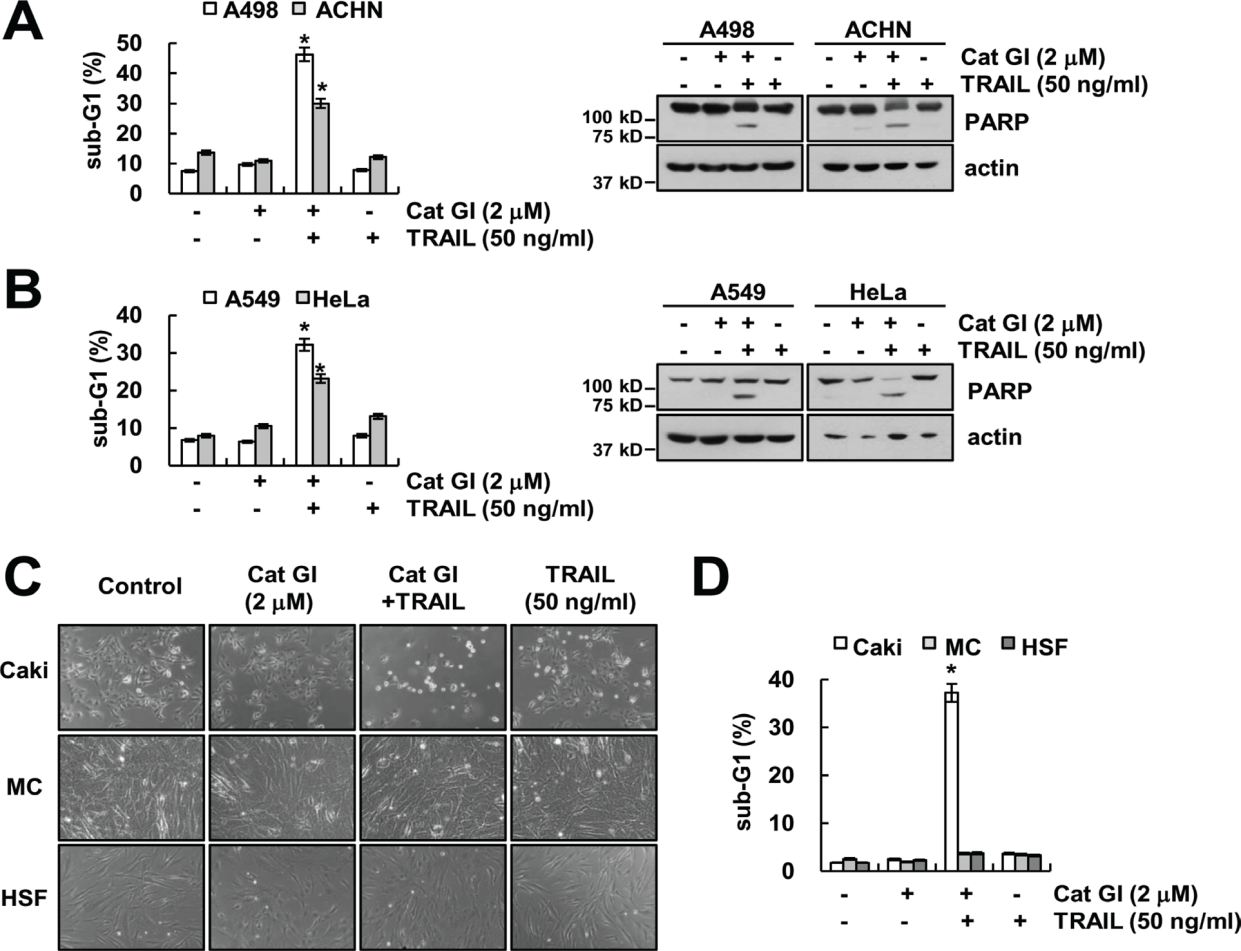
Effect of the combined treatment with Cat GI and TRAIL on apoptosis in other cancer and normal cells (**A**–**B**) Renal carcinoma (A498 and ACHN) (A), lung cancer (A549) and cervical cancer (HeLa) (B) were treated with 50 ng/ml TRAIL in the presence or absence of 2 µM Cat GI for 24 h. The level of apoptosis was analyzed by the sub-G1 population using flow cytometry (left panel). The protein expression levels of PARP and actin were determined by western blotting. The level of actin was used as a loading control (right panel). (**C–D**) Caki, mesangial cell (MC) and human skin fibroblast (HSF) cells were treated with 50 ng/ml TRAIL in the presence or absence of 2 µM Cat GI for 24 h. The cell morphology was examined using interference light microscopy (C). The level of apoptosis was analyzed by the sub-G1 population using flow cytometry (D). The values in A, B and D represent the mean ± SD from three independent samples; ^*^p < 0.01 compared to the control.

Next, we investigated whether combined treatment Cat GI and TRAIL induces apoptosis in other cancer cells and normal cells. As shown in Figure 2A and 2B, we found that combined treatment with Cat GI plus TRAIL increased apoptosis population and PARP cleavage in renal carcinoma cells (ACHN and A498), lung cancer cells (A549) and cervical cancer cells (HeLa). In contrast, Cat GI plus TRAIL had no effect on apoptosis and morphological changes in normal human mesangial cells (MC) and human skin fibroblast (HSF) (Figure 2C and 2D). Taken together, these data suggest that Cat GI could selectively enhance TRAIL-induced apoptosis in cancer cells, but not in normal cells.

### Cat GI induces down-regulation of survivin expression

**Figure 3:**
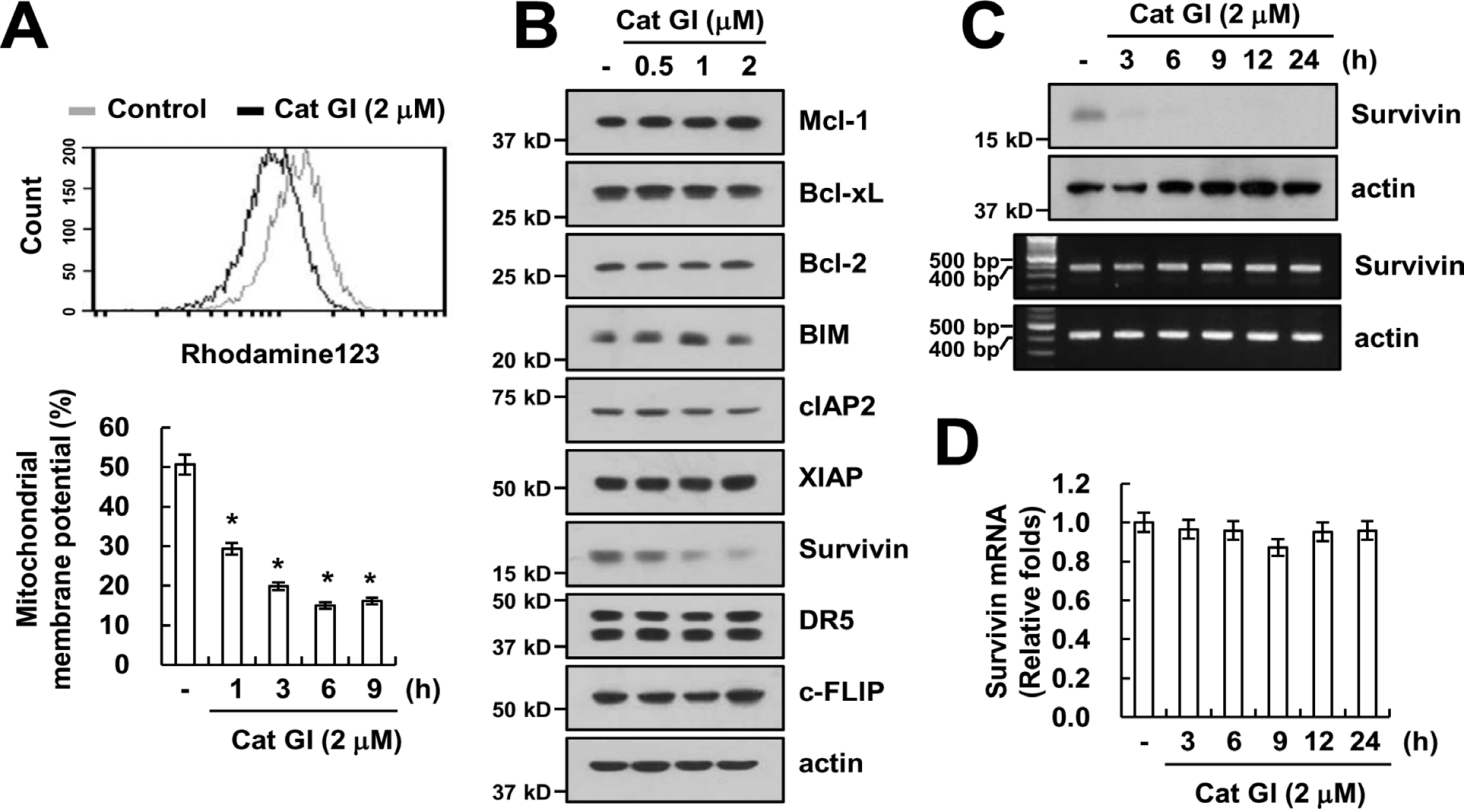
Cat GI induces mitochondria membrane permeability and down-regulation of survivin expression (**A**) Caki cells were treated with 2 µM Cat GI for 3 h (upper panel) or the indicated time periods (lower panel) and loaded with a rhodamine123 fluorescent dye. The mitochondrial membrane potential (MMP) was measured using a flow cytometer. (**B**) Caki cells were treated with the indicated concentrations of Cat GI for 24 h. The protein expression levels of Mcl-1, Bcl-xL, Bcl-2, BIM, cIAP2, XIAP, survivin, DR5, c-FLIP and actin were determined by western blotting. The level of actin was used as a loading control. (**C–D**) Caki cells were treated with 2 µM Cat GI for indicated time periods. The protein and mRNA expression levels of survivin and actin were determined by western blotting (C, upper panel), RT-PCR (C, lower panel) and quantitative PCR (qPCR, D). The level of actin was used as a loading control. The values in A and D represent the mean ± SD from three independent samples; ^*^p < 0.01 compared to the control.

Previous studies reported that loss of mitochondria membrane potential (MMP) plays critical role in overcome of TRAIL resistance [31, 32]. Therefore, we measured the loss of MMP using rhodamine123 fluorescence dye. Cat GI markedly reduced the loss of MMP level (Figure 3A). To identify the molecular mechanisms underlying sensitization to TRAIL by Cat GI, we examined the expression levels of apoptosis-related proteins. As shown in Figure 3B, the expression of protein levels of anti- and pro-apoptotic Bcl-2 family (Mcl-1, Bcl-xL, Bcl-2 and BIM), IAP family (cIAP2 and XIAP), DR5 and c-FLIP did not change by Cat GI, but, Cat GI efficiently decreased survivin expression within 3 h (Figure 3B and 3C). We next examined whether Cat GI regulates survivin expression at the transcriptional level. Cat GI did not alter survivin mRNA expression (Figure 3C and 3D). These data indicate that Cat GI induced loss of MMP level and down-regulation of survivin protein expression.

### Down-regulation of survivin is associated with Cat GI plus TRAIL-induced apoptosis

**Figure 4:**
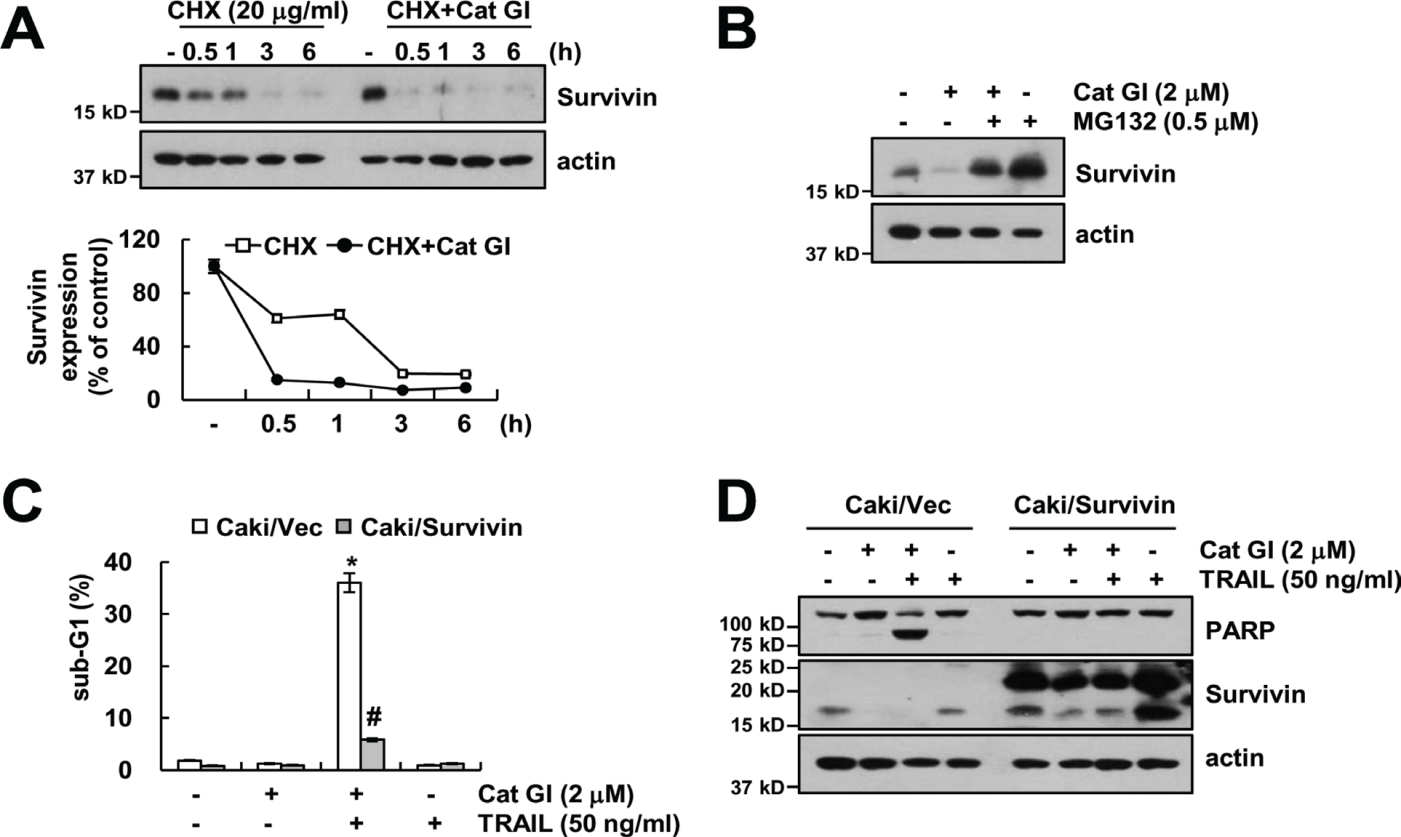
Down-regulation of survivin by Cat GI is involved in the induction of TRAIL-mediated apoptosis (**A**) Caki cells were treated with or without 2 µM Cat GI in the presence of 20 mg/ml cyclohexamide (CHX) for the indicated time periods. The protein expression levels of survivin and actin were determined by western blotting. The level of actin was used as a loading control (upper panel). The band intensity of the survivin protein was measured using the public domain JAVA image-processing program ImageJ (lower panel). (**B**) Caki cells were pretreated with 0.5 µM MG132 for 30 min, and then treated with 2 µM Cat GI for 24 h. The protein expression levels of survivin and actin were determined by western blotting. The level of actin was used as a loading control. (**C–D**) Vector-transfected cells (Caki/Vec) and survivin-overexpressing cells (Caki/Survivin) were treated with 50 ng/ml TRAIL in the presence or absence of 2 µM Cat GI for 24 h. The level of apoptosis was analyzed by the sub-G1 population using flow cytometry (C). The protein expression levels of PARP, survivin and actin were determined by western blotting. The level of actin was used as a loading control (D). The values in C represent the mean ± SD from three independent samples; ^*^p < 0.01 compared to control Caki/Vec. ^#^*p* < 0.01 compared to the Cat GI plus TRAIL-treated Caki/Vec.

Cat GI induced down-regulation of survivin protein expression levels. To further examine the mechanism underlying down-regulation of survivin expression by Cat GI, we examined protein stability of survivin. Caki cells were pretreated with cycloheximide (CHX), an inhibitor of protein synthesis, in the presence or absence of Cat GI. Cat GI plus CHX significantly reduced survivin stability compared with CHX alone (Figure 4A). Because expression of survivin protein is regulated by the ubiquitin-proteasome pathway [33], we checked whether proteasome inhibitor (MG132) reverses Cat GI-mediated down-regulation of survivin expression. MG132 markedly inhibited Cat GI-induced down-regulation of survivin expression (Figure 4B). To identify the significance of the down-regulation of survivin protein, we treated survivin-overexpressing cells with TRAIL in the presence or absence of Cat GI. Overexpression of survivin markedly blocked apoptosis and PARP cleavage in Cat GI plus TRAIL-treated cells (Figure 4C and 4D). Therefore, these results suggest that Cat GI-induced survivin down-regulation is associated with TRAIL sensitization.

### Effect of ROS on Cat GI plus TRAIL-induced apoptosis

**Figure 5:**
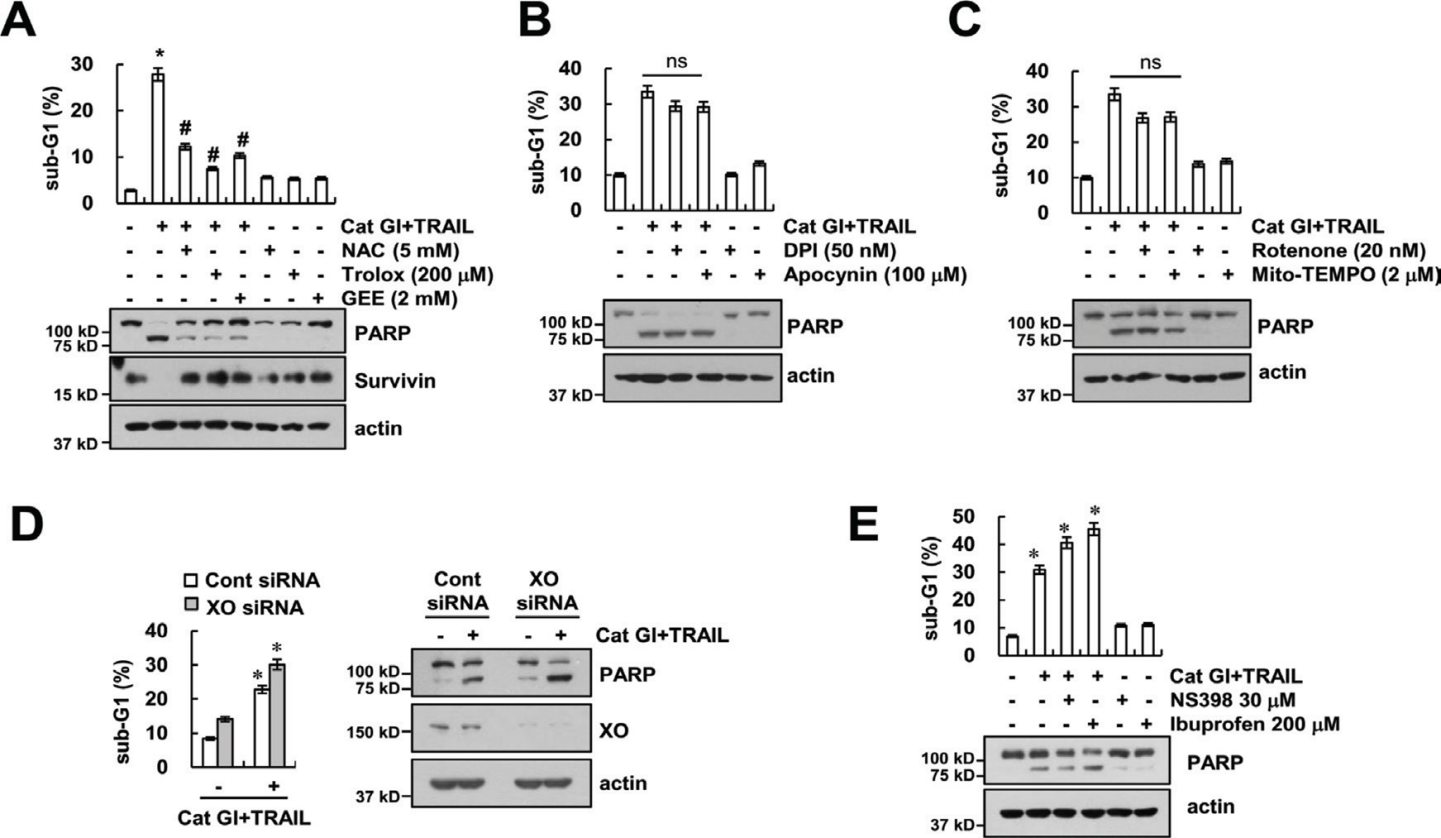
Cat GI plus TRAIL-induced apoptosis is dependent of ROS signaling in Caki cells (**A**) Caki cells were treated with 2 µM Cat GI plus 50 ng/ml TRAIL for 24 h in the presence or absence of 5 mM NAC, 200 µM trolox and 2 mM GEE. (**B**) Caki cells were treated with 2 µM Cat GI plus 50 ng/ml TRAIL for 24 h in the presence or absence of 50 nM DPI and 100 µM apocynin. (**C**) Caki cells were treated with 2 µM Cat GI plus 50 ng/ml TRAIL for 24 h in the presence or absence of 20 nM rotenone and 2 µM Mito-TEMPO. (**D**) Caki cells were transiently transfected control siRNA (Cont siRNA) or xanthine oxidase siRNA (XO siRNA). Twenty-four hours after transfection, cells were treated with 2 µM Cat GI plus 50 ng/ml TRAIL for 24 h. (**E**) Caki cells were pretreated with 30 µM NS398 and 200 µM Ibuprofen for 30 min, and then added with 2 µM Cat GI plus 50 ng/ml TRAIL for 24 h. The level of apoptosis was analyzed by the sub-G1 population using flow cytometry. The protein expression levels of PARP, XO, survivin and actin were determined by western blotting. The values in A, B, C, D, and E represent the mean ± SD from three independent samples; ^*^p < 0.01 compared to control. ^#^*p* < 0.01 compared to the combined treatment with Cat GI and TRAIL. ns (not significant).

The generation of ROS plays a critical role in TRAIL-mediated apoptosis [34–36]. Therefore, we investigated whether ROS signaling is involved in Cat GI plus TRAIL-induced apoptosis. Pretreatment with ROS scavengers [N-acetyl-L-cysteine (NAC), trolox and glutathione ethyl ester (GEE)] markedly inhibited apoptosis and PARP cleavage in combined treatment with Cat GI plus TRAIL (Figure 5A). Furthermore, ROS scavengers reversed Cat GI-mediated down-regulation of survivin expression (Figure 5A). Next, we investigated the source of ROS production in Cat GI-treated cells. ROS are mainly generated by the mitochondrial electron transport chain [37] and NOX [38]. Caki cells were treated with NOX inhibitors [diphenyleneiodonium (DPI) and apocynin], mitochondrial complex I inhibitor (rotenone) and specific mitochondrial superoxide scavenger (Mito-TEMPO). As shown in Figure 5B and 5C, NOX inhibitors or mitochondria ROS inhibitors did not affect apoptosis and PARP cleavage in Cat GI plus TRAIL-treated cells. Furthermore, knockdown of XO expression by siRNA and COX inhibitor, NS398, did not reverse apoptosis and PARP cleavage in Cat GI plus TRAIL-treated cells (Figure 5D and 5E).

### 5-lipoxygenases-derived ROS play a critical role in Cat GI plus TRAIL-induced apoptosis

**Figure 6:**
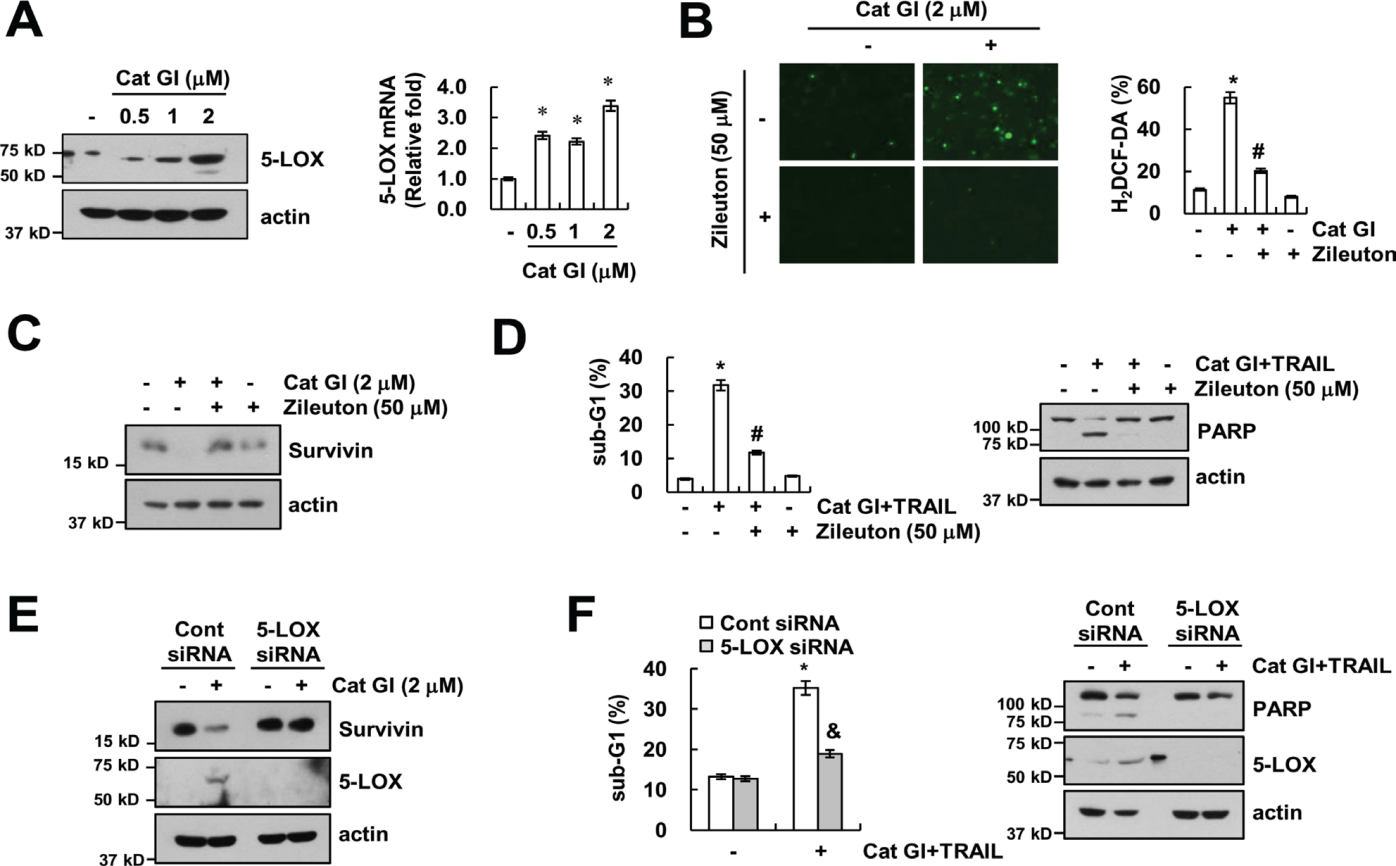
Up-regulation of 5-LOX has a critical role in Cat GI plus TRAIL-induced apoptosis (**A**) Caki cells were treated with the indicated concentrations of Cat GI for 24 h. The protein and mRNA expression levels of 5-LOX and actin were determined by western blotting (left panel) and qPCR (right panel). The level of actin was used as a loading control. (**B**–**C**) Caki cells were treated with 2 µM Cat GI for 2 h (B) or 24 h (C) in the presence or absence of 50 µM zileuton. The cells were then loaded with the H_2_DCF-DA fluorescent dye. The H_2_DCF-DA fluorescence intensity was detected by a fluorescence microscope (B, left panel) and flow cytometry (B, right panel). The protein expression levels of 5-LOX and actin were determined by western blotting. The level of actin was used as a loading control (C). (**D**) Caki cells were treated with 2 µM Cat GI plus 50 ng/ml TRAIL for 24 h in the presence or absence of 50 µM zileuton. The level of apoptosis was analyzed by the sub-G1 population using flow cytometry. The protein expression levels of PARP and actin were determined by western blotting. The level of actin was used as a loading control. (**E**) Caki cells were transiently transfected control siRNA (Cont siRNA) or 5-LOX siRNA. Twenty-four hours after transfection, cells were treated with 2 µM Cat GI for 24 h. The protein expression levels of survivin, 5-LOX and actin were determined by western blotting. The level of actin was used as a loading control. (**F**) Caki cells were transiently transfected control siRNA (Cont siRNA) or 5-LOX siRNA. Twenty-four hours after transfection, cells were treated with 2 µM Cat GI plus 50 ng/ml TRAIL for 24 h. The level of apoptosis was analyzed by the sub-G1 population using flow cytometry. The protein expression levels of PARP, 5-LOX and actin were determined by western blotting. The level of actin was used as a loading control. The values in A, B, D, and F represent the mean ± SD from three independent samples; ^*^p < 0.01 compared to the control. ^#^*p* < 0.01 compared to the Cat GI plus TRAIL. ^&^*p* < 0.01 compared to the Cat GI plus TRAIL-treated control siRNA.

The production of intracellular ROS is also induced by 5-LOX [39]. Therefore, we examined the expression levels of 5-LOX protein and mRNA in Cat GI-treated cells. As shown in Figure 6A, Cat GI up-regulated 5-LOX protein and mRNA expression in dose-dependent manner (Figure 6A). To investigate whether 5-LOX is involved in Cat GI-induced ROS production, we used zileuton, a 5-LOX inhibitor. Zileuton markedly inhibited Cat GI-induced ROS production and down-regulation of survivin expression (Figure 6B and 6C). In addition, zileuton markedly reduced apoptosis by combined treatment with Cat GI and TRAIL (Figure 6D). To further confirm the importance of 5-LOX on Cat GI-mediated survivin down-regulation, we employed 5-LOX siRNA. Down-regulation of 5-LOX expression by siRNA markedly reversed the down-regulation of survivin expression in Cat GI-treated cells (Figure 6E). In addition, knockdown of 5-LOX prevented apoptosis and PARP cleavage in Cat GI plus TRAIL-treated cells (Figure 6F). Therefore, these data suggest that 5-LOX-induced ROS generation plays a critical role in Cat GI-mediated survivin down-regulation and TRAIL sensitization.

### Knockdown of cathepsin G sensitizes TRAIL-mediated apoptosis

**Figure 7:**
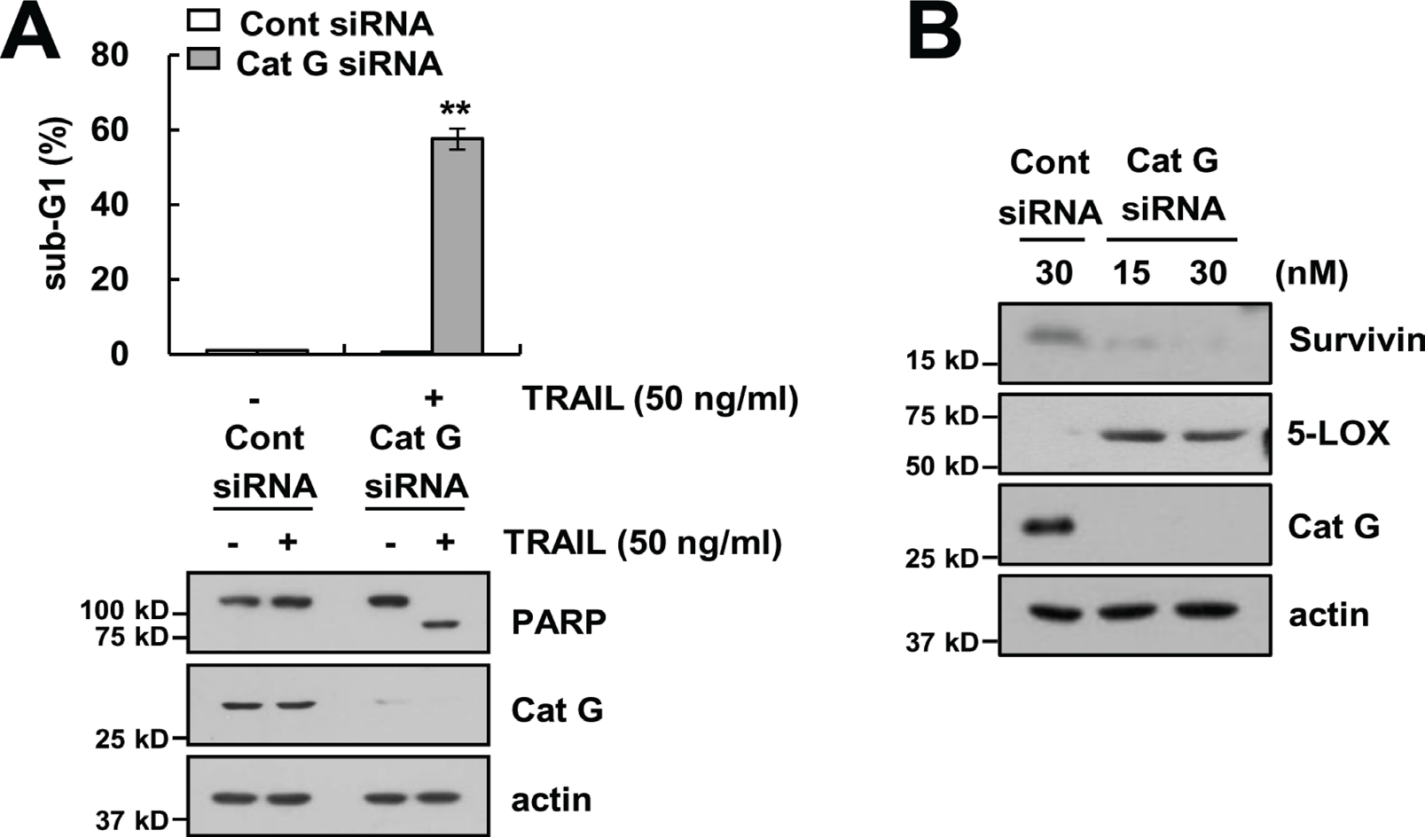
Knockdown of cathepsin G sensitizes Caki cells to TRAIL-mediated apoptosis (**A**) Caki cells were transiently transfected control siRNA (Cont siRNA) or cathepsin G siRNA (Cat G siRNA). Twenty-four hours after transfection, cells were treated with 50 ng/ml TRAIL for 24 h. The level of apoptosis was analyzed by the sub-G1 population using flow cytometry. The protein expression levels of PARP, cathepsin G and actin were determined by western blotting. The level of actin was used as a loading control. (**B**) Caki cells were transiently transfected Cont siRNA or Cat G siRNA for 24 h. The protein expression levels of survivin, 5-LOX, Cat G and actin were determined by western blotting. The level of actin was used as a loading control. The values in A represent the mean ± SD from three independent samples; ^**^p < 0.01 compared to the TRAIL-treated control siRNA.

We investigated whether the effect of Cat GI on TRAIL sensitization is dependent on the inhibition of cathepsin G. Caki cells were transiently transfected with cathepsin G siRNA. Knockdown of cathepsin G by siRNA markedly sensitized TRAIL-mediated apoptosis and increased PARP cleavage (Figure 7A). We also found that knockdown of cathepsin G by siRNA induced survivin down-regulation and increased 5-LOX expression (Figure 7B). Therefore, these results indicate that both genetic and pharmacological inhibition of cathepsin G could sensitize Caki cells to TRAIL-mediated apoptosis through 5-LOX-dependent down-regulation of survivin expression.

## DISCUSSION

In this study, we found that inhibition of cathepsin G enhances TRAIL-mediated apoptosis in cancer cells but not in normal cells. Inhibition of cathepsin G induced down-regulation of survivin expression at the post-translational level. In this event, 5-LOX-dependent ROS generation played an important role in survivin down-regulation and TRAIL sensitization.

Survivin is the smallest protein of IAP family and then inhibited apoptosis through inactivation of caspases. It is overexpressed in many cancer cells including breast cancer [40], pancreatic cancer [41] and osteosarcoma [42]. Expression of survivin is modulated by transcriptional and post-translational levels. Cat GI and cathepsin G siRNA induced down-regulation of survivin protein through decrease of protein stability (Figures 3B, 4A, and 8B). Post-translational regulation of survivin was modulated by variety molecular mechanisms. 1) Phosphorylation of survivin (Thr34) by cdc2 increases stability of protein expression [43], and inhibition of phosphorylation at Thr34 induced degradation of survivin [44, 45]. 2) Complex formation of the survivin-Hsp90 increases survivin protein stability [46]. Disruption of the survivin-Hsp90 complex by Hsp90 inhibitor promotes degradation of survivin, resulted in apoptosis in cervical carcinoma HeLa cells [46]. 3) Survivin-aryl hydrocarbon receptor-interacting protein (AIP) complex also increases survivin protein stability [47]. Kang *et al.* identify the novel survivin binding protein, AIP using proteomic screening. AIP directly bind to survivin, and down-regulation of AIP by siRNA specifically reduces survivin protein stability, but not XIAP [47]. 4) E3 ligase of survivin modulates protein expression. For example, Arora *et al.* reported that activation of E3 ligase by complex of XIAP-XAF1 is associated with the proteasome-dependent survivin down-regulation [48]. Increase of FBXL7, SCF ubiquitin E3 ligase component, induces ubiquitylation and degradation of survivin in gastric cancer [49]. Multiple mechanisms are related with modulation of survivin protein stability. Therefore, we need further study to identify mechanisms of cathepsin G-mediated survivin degradation.

ROS are generated in mitochondria electron transport chain and cytosol enzymes (NOX, XO, LOX, and COX). In our study, Cat GI inhibited survivin expression and induced TRIAL-mediated apoptosis in an ROS-dependent manner (Figure 5A). However, both NOX inhibitor and mitochondrial superoxide scavenger (Mito-TEMPO) did not block combined treatment with Cat GI plus TRAIL-induced apoptosis and down-regulation of survivin (Figure 5B and 5C). Furthermore, knockdown of XO expression by siRNA and COX inhibitor did not reverse apoptosis in Cat GI plus TRAIL-treated cells (Figure 5D and 5E). Thus, these data indicate that XO and COX are not involved in Cat GI-induced TRAIL sensitization.

5-LOX has been shown to be critical in mediating oxidative stress [50]. In previous studies, inhibition of 5-LOX induced inhibition of proliferation [51] and down-regulation of XIAP in human glioblastoma cells [52]. Interestingly, our data indicate that genetic (siRNA) and pharmacological inhibition of 5-LOX (zileuton) markedly blocked combined treatment with Cat GI plus TRAIL-induced apoptosis (Figure 6D and 6F). Zileuton inhibited Cat GI-induced ROS generation and survivin down-regulation (Figure 6B and 6C). Therefore, these results suggest that 5-LOX plays a major role in Cat GI plus TRAIL-mediated apoptosis.

Collectively, these findings supported that inhibition of cathepsin G induces 5-LOX mediated ROS that sensitizes TRAIL-induced apoptotic cell death through down-regulation of survivin in human renal Caki cells. Therefore, the combination of cathepsin G inhibition and TRAIL could be a therapeutic strategy for cancer therapy.

## MATERIALS AND METHODS

### Cell cultures and materials

Human renal carcinoma (Caki, ACHN and A498), human lung cancer (A549) and human cervical cancer (HeLa) were obtained from the American Type Culture Collection (Manassas, VA, USA). Primary culture of human mesangial cells (Cryo NHMC) were purchased from Clonetics (San Diego, CA, USA). The human skin fibroblast (HSF) was a gift from Dr. T.J. Lee (Yeungnam University, Korea). All cells were cultured in Dulbecco’s Modified Eagle’s Medium containing 10% fetal bovine serum, 20 mM HEPES buffer, 100 U/ml penicillin, 100 mg/ml streptomycin, and 100 mg/ml gentamicin. The PCR primers were purchased from Macrogen (Seoul, Korea). Cathepsin G inhibitor I (Cat GI), anti-Mcl-1, anti-cIAP2 and anti-cathepsin G antibodies were purchased from Santa Cruz Biotechnology (Dallas, TX, USA). Recombinant human TRAIL, z-VAD-fmk and anti-survivin antibody were purchased from R&D system (Minneapolis, MN, USA). N-acetyl-L-cysteine (NAC), trolox, NS398 were obtained from Calbiochem (San Diego, CA, USA). Anti-Bim and anti-XIAP antibodies were purchased from BD Biosciences (San Jose, CA, USA). Anti-PARP, anti-Bcl-2, anti-Bcl-xL and anti-DR5 antibodies were purchased from Cell Signaling Technology (Beverly, MA, USA). Anti-c-FLIP antibody was obtained from Enzo life science (Farmington, NY, USA). Anti-5-LOX antibody and Ibuprofen were purchased from Abcam (Cambridge, MA, USA). Anti-actin antibody and other chemicals were purchased from Sigma Chemical Co. (St. Louis, MO, USA).

### Flow cytometry analysis

For flow cytometry, the cells were resuspended in 100 μl of phosphate-buffered saline (PBS), and 200 μl of 95% ethanol was added while the cells were being vortexed. Then, the cells were incubated at 4°C for 1 h, washed with PBS, resuspended in 200 μl of 1.12% sodium citrate buffer (pH 8.4) with 12.5 μg of RNase and incubated for an additional 30 min at 37°C. The cellular DNA was then stained by adding 200 μl of a propidium iodide solution (50 μg/ml) to the cells for 30 min at room temperature. The stained cells were analyzed by fluorescent-activated cell sorting on a FACScan flow cytometer (BD Biosciences, San Jose, CA, USA) to determine the relative DNA content, which was based on the red fluorescence intensity.

### Western blot analysis

Cells were washed with cold PBS and lysed on ice in 50 µL of lysis buffer (50 mM Tris-HCl, 1 mM EGTA, 1% Triton X-100, 1 mM phenylmethylsulfonyl fluoride, pH 7.5) [53, 54]. Lysates were centrifuged at 10,000 × g for 15 min at 4°C, and the supernatant fractions were collected. Proteins were separated by SDS-PAGE and transferred to an Immobilon-P membrane (GE Healthcare Life Science, Pittsburgh, PO, USA). Specific proteins were detected using an enhanced chemiluminescence (ECL) western blot kit (EMD Millipore, Darmstadt, Germany) according to the manufacturer’s instructions.

### 4′,6′-Diamidino-2-phenylindole staining (DAPI) for nuclei condensation and fragmentation

To examine cellular nuclei, the cells were fixed with 1% paraformaldehyde on glass slides for 30 min at room temperature. After the fixation, the cells were washed with PBS and a 300 nM DAPI solution (Roche, Basel, Switzerland) was added to the fixed cells for 5 min. After the nuclei were stained, the cells were examined by fluorescence microscopy (Carl Zeiss, Jena, Germany).

### Cell death assessment by DNA fragmentation assay

The cell death detection ELISA plus kit (Boehringer Mannheim, Indianapolis, IN, USA) was used for assessing apoptotic activity by detecting fragmented DNA within the nucleus in Cat GI-, TRAIL-, and combination with Cat GI and TRAIL-treated cells. Briefly, each culture plate was centrifuged for 10 min at 200 × g, the supernatant was removed, and the pellet was lysed for 30 min. After centrifuging the plate again at 200 × g for 10 min, and the supernatant that contained the cytoplasmic histone-associated DNA fragments was collected and incubated with an immobilized anti-histone antibody. The reaction products were incubated with a peroxidase substrate for 5 min and measured by spectrophotometry at 405 and 490 nm (reference wavelength) with a microplate reader (BMG Labtech, Ortenberg, Germany). The signals in the wells containing the substrate alone were subtracted as the background.

### Asp-Glu-Val-Asp-ase (DEVDase) activity assay

To evaluate DEVDase activity, cell lysates were prepared after their respective treatments with TRAIL in the presence or absence of Cat GI. Assays were performed in 96-well microtiter plates by incubating 20 μg of cell lysates in 100 ml of reaction buffer (1% NP-40, 20 mM Tris-HCl, pH 7.5, 137 mM NaCl, 10% glycerol) containing a caspase substrate [Asp-Glu-Val-Asp-chromophore-p-nitroanilide (DEVD-pNA)] at 5 µM. Lysates were incubated at 37°C for 2 h. Thereafter, the absorbance at 405 nm was measured with a spectrophotometer (BMG Labtech, Ortenberg, Germany).

### Determination of synergy

The possible synergistic effect of Cat GI and TRAIL was evaluated using the isobologram method. In brief, cells were treated with different concentrations of Cat GI and TRAIL alone or in combination. After 24 h, relative survival was assessed, and the concentration effect curves were used to determine the IC50 (the half-maximal inhibitory concentration) values for each drug alone and in combination with a fixed concentration of the second agent.

### Reverse transcription polymerase chain reaction (RT-PCR) and quantitative PCR (qPCR)

Total RNA was isolated using the TriZol reagent (Life Technologies, Gaithersburg, MD, USA), and the cDNA was prepared using M-MLV reverse transcriptase (Gibco-BRL, Gaithersburg, MD, USA) according to the manufacturer′s instructions. The following primers were used for the amplification of human survivin and actin: survivin (forward) 5′-GGA CCA CCG CAT CTC TAC AT-3′ and (reverse) 5′-GCA CTT TCT TCG CAG TTT CC-3′; and actin (forward) 5′-GGC ATC GTC ACC AAC TGG GAC-3′ and (reverse) 5′-CGA TTT CCC GCT CGG CCG TGG-3′. The PCR amplification was carried out using the following cycling conditions: 94°C for 3 min followed by 17 (actin) or 30 cycles (survivin) of 94°C for 40 s, 56°C for 40 s, 72°C for 1 min, and a final extension at 72°C for 5 min. The amplified products were separated by electrophoresis on a 1.5% agarose gel and detected under UV light. For qPCR, cDNA and forward/reverse primers (200 nM) were added to 2 × KAPA SYBR Fast master mix, and reactions were performed on LightCycler 480 real-time amplification instrument (Roche, Basel, Switzerland). The following primers were used for the amplification of human survivin and actin: survivin (forward) 5′-TTC TCA AGG ACC ACC GCA TC-3′ and (reverse) 5′-GTT TCC TTT GCA TGG GGT CG-3′; and actin (forward) 5′-CTA CAA TGA GCT GCG TGT G-3′ and (reverse) 5′-TGG GGT GTT GAA GGT CTC-3′. Threshold cycle number (Ct) of each gene was calculated, and actin was used as reference genes. Delta-delta Ct values of genes were presented as relative fold induction.

### Measurement of mitochondrial membrane potential by rhodamine 123

Rhodamine 123 (Molecular Probes Inc., Eugene, OR) uptake by mitochondria is directly proportional to its membrane potential. After treatment, cells were incubated with rhodamine 123 (5 µM) for 5 min in the dark at 37°C. The cells were harvested and suspended in PBS. The mitochondrial membrane potential was subsequently analyzed using a flow cytometer.

### Stable transfection in Caki cells

The Caki cells were transfected in a stable manner with the pcDNA3.1/survivin-flag or control plasmid pcDNA 3.1 vector using Lipofectamine™ 2000 as prescribed by the manufacturer (Invitrogen, Carlsbad, CA, USA). After 48 h of incubation, transfected cells were selected in primary cell culture medium containing 700 μg/mL G418 (Invitrogen, Carlsbad, CA, USA). After 2 or 3 weeks, single independent clones were randomly isolated, and each individual clone was plated separately. After clonal expansion, cells from each independent clone were tested for survivin expression by immunoblotting.

### Measurement of ROS

Intracellular accumulation of ROS was determined using the fluorescent probes 2′,7′-dichlorodihydrofluorescein diacetate (H_2_DCFDA). Caki cells were treated with Cat GI, and then cells were stained with the H_2_DCFDA fluorescent dye for an additional 10 min. Then, cells were trypsinized and resuspended in PBS, and fluorescence was measured at specific time intervals with a flow cytometer (BD Biosciences, San Jose, CA, USA) or fluorescence microscope (Carl Zeiss, Jena, Germany).

### Small interfering RNA (siRNA)

The 5-LOX siRNA, cathepsin G siRNA, and xanthine oxidase siRNA used in this study were purchased from Santa Cruz Biotechnology (Dallas, TX, USA). The GFP (control) siRNA was purchased from Bioneer (Daejeon, Korea). Cells were transfected with siRNA oligonucleotides using Lipofectamine^®^ RNAiMAX Reagent (Invitrogen, Carlsbad, CA, USA) according to the manufacturerʼs recommendations.

### Statistical analysis

The data were analyzed using a one-way ANOVA and post-hoc comparisons (Student-Newman-Keuls) using the Statistical Package for Social Sciences 22.0 software (SPSS Inc., Chicago, IL, USA).

## Abbreviations

Nox: NADPH oxidase
ROS: Reactive oxygen species
CHX: cycloheximide
Cat GI: Cathepsin G inhibitor I
5-LOX: 5-lipoxygenase
TNF: Tumor necrosis factor
TRAIL: TNF-related apoptosis-inducing ligand
XO: xanthine oxidases
COX: cyclooxygenases
NAC: N-acetyl-L-cysteine
GEE: glutathione ethyl ester
